# Rupture of an acute ascending aortic dissection causing right pulmonary artery occlusion

**DOI:** 10.1259/bjrcr.20210043

**Published:** 2022-03-09

**Authors:** Priyesh Karia, Ayyaz Quddus, Kesavan Nayagam, Olga Lazoura

**Affiliations:** 1 Royal Free London NHS Foundation Trust, London, UK

## Abstract

Rupture of ascending thoracic aortic dissection mimicking pulmonary thromboembolism due to pulmonary artery occlusion is rare and should be considered in hypoxic patients with aortic dissection.

## Imaging findings

**Figure 1.**
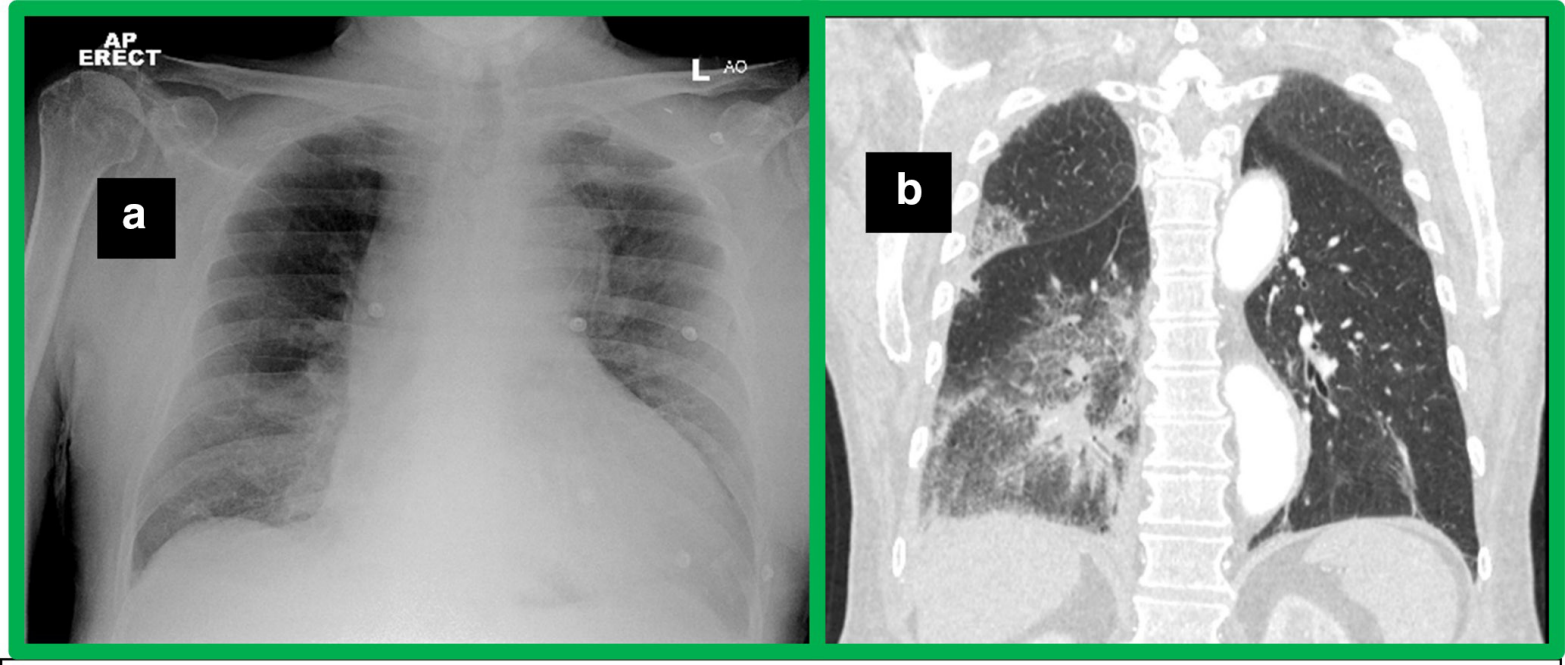
a. AP chest radiograph demonstrates airspace opacification in the right lung mainly involving the mid and lower lung zones. b. Lung window coronal reconstruction of ECG-gated CT aortogram confirms extensive ground glass change in the right lung more pronounced in the lower lobe. AP, anteroposterior; ECG-gated CT, electrocardiographically gated CT.

We describe a case of an 88-year-old male who presented with shortness of breath and hypoxia. Chest radiograph demonstrated airspace opacification in the right lung mainly involving the mid and lower zones (Figure 1a). Transthoracic echocardiogram (TTE) showed an ascending aortic dissection flap and the patient proceeded to electrocardiographically gated CT aortography.

**Figure 2.**
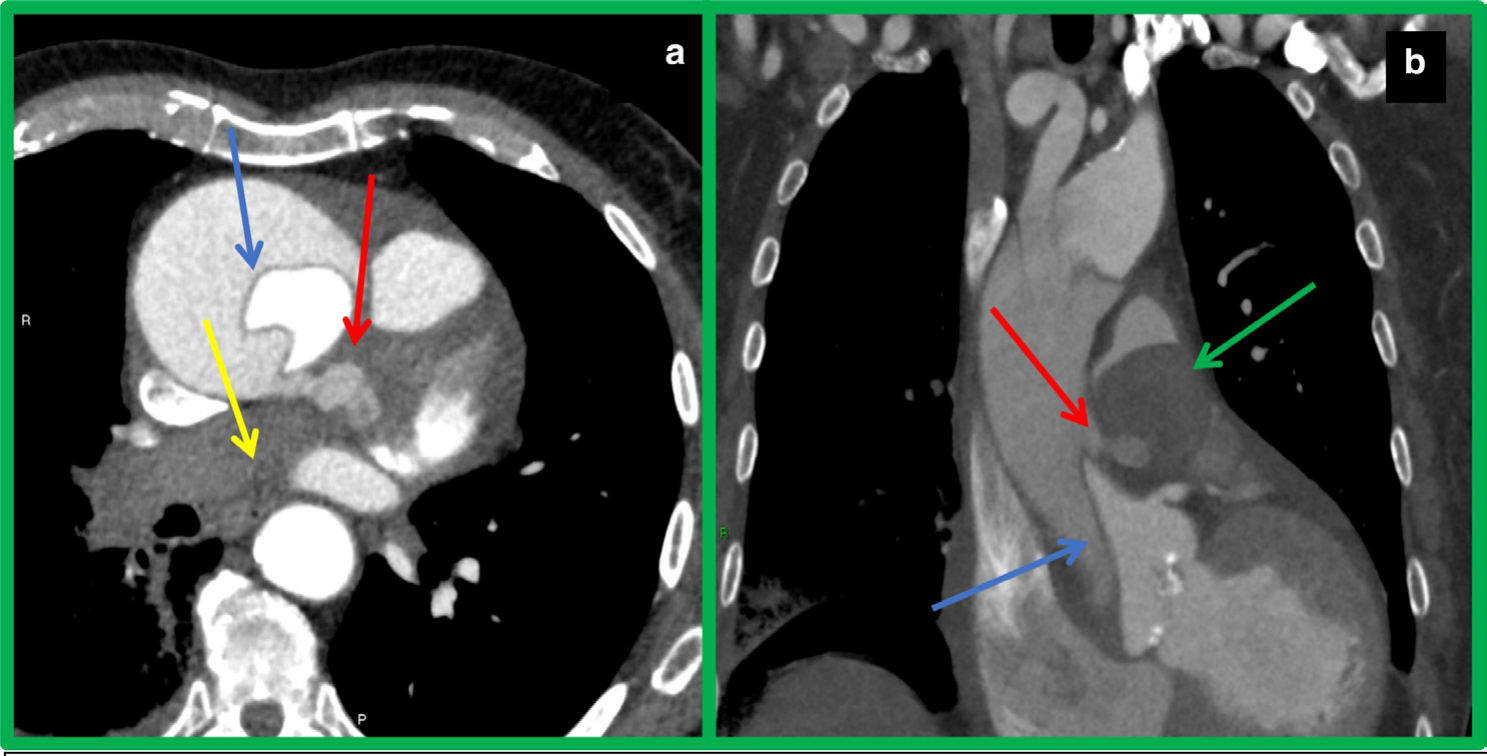
ECG-gated CT aortogram a. axial and b. coronal reconstructions. The ascending thoracic aorta is dilated with a dissection flap (blue arrow) extending to the aortic root. Acute rupture of the false lumen with contrast extravasation posteriorly into the common sheath of the ascending aorta and PT (red arrow) is causing a large haematoma (green arrow) compressing the PT and proximal RPA. Lack of contrast opacification of the RPA (yellow arrow) and haemotoma (green arrow) represent external compression at the bifurcation of the PT and origin of the RPA, with subsequent no distal filling into the RPA branches. ECG-gated, electrocardiographically gated CT; PT, pulmonary trunk; RPA, right pulmonary artery.

CT demonstrated a dissection flap at the ascending thoracic aorta extending caudally to the aortic root and cranially to the origin of left common carotid artery (Figure 2b). The brachiocephalic trunk originated from the false lumen, the coronary arteries originated from the true lumen. At the level of the pulmonary trunk (PT), there was contrast extravasation from the false lumen posteriorly into the common sheath shared by the ascending aorta and the PT resulting in a localised haematoma (Figure 2). CT demonstrated an apparent large ‘filling defect’ of the PT in continuity with complete lack of contrast opacification of the right pulmonary artery (RPA) and its branches (Figure 2a). Extensive ground glass change in the right lung, more pronounced in the lower lobe, was seen on lung window (Figure 1b). These imaging findings were due to compression of the PT and occlusion at the origin of the RPA caused by the common sheath haematoma and resulting in right pulmonary infarction.

Unfortunately, the patient deteriorated soon after the CT scan and passed away within 24 h after the CT.

## Discussion

The ascending thoracic aorta and the PT are invested in a common sheath of serous visceral pericardium. Occlusion of the RPA from rupture of ascending aortic dissection into the common sheath shared by the aorta and pulmonary artery is rare. It can mimic pulmonary thromboembolism and it is important to distinguish from this as anticoagulation is contraindicated.

One case of an ascending aortic dissecting aneurysm causing RPA occlusion described by De Silva et al^1^ had similar imaging findings mimicking thromboembolism. They describe the susceptibility of compression of the RPA by pathology of the ascending aorta due to the anatomical relationship with the aorta at this level and the common tunica adventitia shared by the aorta and PT. Buja et al^2^ described medial rupture of an ascending aortic aneurysm resulting in adventitial haematoma compressing the PT. A case of pulmonary hypertension caused by compression of the pulmonary arteries by dissecting ascending aortic haematoma was reported by Kim et al^3^ Another case of transient pulmonary hypertension caused by extrinsic compression of the pulmonary trunk was reported by Okiwelu et al^4^, due to a contained rupture of proximal ascending aorta, likely from a penetrating atherosclerotic ulcer.

## Learning points

- It is important to consider rupture of ascending aortic dissection into the common sheath as a cause of pulmonary artery occlusion. Occlusion can mimic pulmonary thromboembolism and it is important to distinguish from this, as anticoagulation is contraindicated.
- CT should be considered in cases of dissection, even when a dissection flap is clearly visible on TTE. CT will demonstrate extension of the dissection in the ascending aorta, aortic root and possible involvement of pulmonary artery, which cannot be assessed on TTE.
